# Assessment of Structural Glaucoma Progression

**DOI:** 10.5005/jp-journals-10008-1108

**Published:** 2012-08-16

**Authors:** Atsuya Miki

**Affiliations:** 1 Hamilton Glaucoma Center, University of California, San Diego

**Keywords:** Glaucoma progression, Retinal nerve fiber layer imaging, Optical coherence tomography, Confocal scanning laser ophthalmoscopy, Scanning laser polarimetry.

## Abstract

**Purpose:**

To provide an update on the role of optic nerve head and peripapillary retinal nerve fiber layer imaging in monitoring glaucoma progression.

**Methods:**

Review of literature.

**Results:**

Imaging technologies, such as optical coherence tomography, scanning laser polarimetry, and confocal scanning laser ophthalmoscopy, objectively and quantitatively measure the structural change associated with glaucoma. Rates of retinal nerve fiber layer (RNFL) and rim area loss are significantly faster in progressing compared with nonprogressing subjects. A number of strategies to detect progression have been proposed. The precision of these methods is generally high. However, there is no agreement as to which instrument or parameter is most appropriate for the evaluation of structural progression associated with glaucoma at this moment. The agreement between structural and functional glaucoma progression is generally poor regardless of the strategies used. Structural progression analyses appear to complement visual field progression analyses, detecting a different subset of progressing subjects.

**Summary:**

Imaging devices are promising tools for monitoring patients with glaucoma. Combining structural and functional analyses is useful for accurate monitoring of glaucoma progression.

**How to cite this article:**

Miki A. Assessment of Structural Glaucoma Progression. J Current Glau Prac 2012;6(2):62-67.

## INTRODUCTION

Glaucoma is an irreversible and progressive optic neuropathy resulting in a characteristic visual field (VF) loss.^1^ Current goal of glaucoma management is to sufficiently decelerate disease progression in order to keep visual function throughout the patient’s life, by controlling intraocular pressure. Assessment of disease progression is, therefore, essential for clinicians to make a decision on whether to initiate or modify glaucoma treatment.

Automated perimetry has been a standard but suboptimal method for assessing glaucoma progression. VF measurement is subject to a variety of influences such as long-term fluctuation, measurement variability and patients’ uncoop-erativeness, all of which may make longitudinal analysis of VF measurements difficult or inaccurate.^2^ Moreover, glaucomatous change in the optic disk may precede detectable VF defects by up to several years.^3-6^

For example, changes in the optic nerve were seen in more than half of the patients before development of VF defects in OHTS study.^5^ In a longitudinal study of ocular hypertensive patients, Sommer et al found retinal nerve fiber layer (RNFL) defects on red-free photographs up to 6 years before the development of VF loss.^3^ A recent study showed that structural changes observed on optic disk stereophotographs are predictive of future functional losses.^7^ These studies clearly demonstrate the necessity and the usefulness of structural evaluation in glaucoma management, especially in its early stage. However, structural assessment was conventionally performed by subjective assessment of color stereophoto-graphs or red-free photographs. These methods depend on clinicians’ subjective interpretation, and hence, have low interobserver agreement.^8^

Recent advancement of imaging technology enabled us to objectively and quantitatively evaluate structural changes associated with glaucoma. Some of these new imaging instruments; scanning laser polarimetry (SLP), confocal scanning laser ophthalmoscopy (CSLO) and optical coherence tomography (OCT); have proven to be useful for diagnosing glaucoma.^9-11^ More recently, analyses of structural glaucoma progression using these new imaging devices have been extensively studied.

In this review, the author will summarize the current knowledge with regard to the features of new imaging devices and the detection of structural glaucoma progression with these devices. Relevant approaches to structural glaucoma progression analysis and their possible advantages and disadvantages will also be discussed.

## STRATEGY TO DETECT PROGRESSION: EVENT ANALYSIS *VS* TREND ANALYSIS

Several strategies to detect structural glaucoma progression have been developed. Most of these methods can be categorized as event-based or trend-based analyses. In event-based analyses, a criterion for change compared with baseline is defined for each parameter, usually based on a statistical calculation of confidence interval for test-retest variability. Event analyses identify progression when a measurement exceeds a predetermined criterion for change (or an event). In contrast, trend-based analyses use linear or other forms of regression analyses to estimate rates of change in parameters. Progression is defined when a statistically significant negative trend is detected.

Trend analyses have an advantage over event analyses in that a rate of change can be estimated. This is particularly useful at the earliest stages of the disease process. A measured rate of progression may assist the assessment of a patient’s risk of development of functionally significant visual loss and in the decision to commence or alter treatment. The major disadvantage of trend analyses is the length of follow-up required to detect progression. The minimal follow-up required to detect progression is influenced by the degree of measurement variability. Long follow-up time and measurement instrument with high reproducibility are the absolute prerequisites for accurate trend analyses.

## SUMMARY OF THE CURRENT EVIDENCE

### Time-domain Optical Coherence Tomography (TD-OCT)

Currently, OCT is the most widely used imaging device for posterior ocular structures including retina and optic nerve head (ONH). OCT is a noninvasive and high-resolution imaging technique based on the principle of low coherence interferometry,^12^ which has been used increasingly to evaluate and manage a variety of retinal diseases. In glaucoma, the RNFL thickness measured by OCT enables an objective and quantitative assessment of glaucomatous structural loss.^10^

Wollstein et al first reported the use of OCT to evaluate structural glaucoma progression using prototype OCT.^13^ They longitudinally evaluated peripapillary RNFL thickness measurements and compared them with standard automated perimetry, using event-based approach. After a median follow-up of 4.7 years, 22% of studied eyes progressed by OCT alone and 9% progressed by SAP alone, and only 3% progressed by both VF and OCT.

Medeiros et al reported results of trend-based analysis of RNFL thickness, optic nerve topography and macular retinal thickness measured with Stratus OCT (Carl Zeiss Meditec).^14^Mean rates of change in average RNFL thickness were significantly higher for progressors compared with nonprogressors (- 0.72 μm/y *vs* 0.14 μm//y; p = 0.004). They also reported that RNFL parameters performed significantly better than ONH and macular thickness measurements in discriminating progressors from nonprogressors.

Lee et al reported the diagnostic ability of Stratus OCT RNFL measurement to discriminate eyes with progressive RNFL atrophy from stable eyes. They reported that the sensitivity and specificity considerably differs depending on what parameter was used; the sensitivity ranged from 14.8% to 85.2%, whereas the specificity ranged from 59.7% to 95%.^15^

Leung et al reported the poor agreement between the trend analysis of RNFL thickness measured by Stratus OCT and the trend analysis of SAP visual field index (VFI). Of 116 eyes examined, 21 eyes showed progression by RNFL, 22 progressed by VFI, but only 3 eyes showed progression by both measures. Another significant finding is that an eye with greater baseline RNFL thickness had higher rate of progression. They also report the poor agreement among three instrument for progression detection [Stratus OCT, SAP and confocal scanning laser ophthalmoscopy (CSLO)] which showed the k value ranging from 0.10 to 0.13.^17^

Lee et al recently reported the ability of trend-based RNFL thickness analysis to discriminate eyes with progressive RNFL thinning from stable eyes using Stratus OCT.^18^ They reported a sensitivity of 62% at a specificity of >80%. However, we should take into consideration the possibility of overestimating agreement because they selectively enrolled patients with localized defect.

### Spectral-domain Optical Coherence Tomography (SD-OCT)

Recently, a new generation of the technology; spectral domain (SD) OCT, which improved the scanning speed and resolution dramatically compared to the conventional time domain (TD)-OCT system, has become available. At this point, there is only one report on the analysis of glaucomatous structural progression using SD-OCT.^19^ The performances of SD-OCT (Cirrus HD-OCT, Carl Zeiss Meditec) and TD-OCT (Stratus OCT) to detect RNFL thickness change were studied in this report. The agreement between these instruments was very poor (κ = 0.188). SD-OCT outperformed TD-OCT in detecting more eyes with RNFL progression and fewer eyes with RNFL improvement.

Different from TD-OCT, SD-OCT extracts circumpapil-lary RNFL thickness measurement data from a cube scan. This method enables the serial RNFL thickness measurements at the same location, thereby reducing measurement variability. Smaller variability may lead to more accurate analyses with fewer follow-up. Another potential advantage of SD-OCT is its ability to measure thicknesses of macular inner retinal layers such as ganglion cell complex or ganglion cell layer.^20-22^ However, more research is necessary to establish the usefulness of SD-OCT in assessing structural progression in glaucoma.

### Scanning Laser Polarimetry

Scanning laser polarimetry (SLP) is based on the principle that polarized light passing through a birefringent medium undergoes retardation proportional to the thickness of the medium.^23^ Since the RNFL consists of retinal ganglion cell axons containing microtubules arranged in parallel, the RNFL exhibits birefringence. In 1990, Weinreb et al described the prototype of the SLP obtaining polarization data from two fixed monkey eyes.^24^ An excellent correlation was found between retardation and histopathologic RNFL measurements. In clinical practice, SLP is performed by a confocal scanning laser ophthalmoscope with an integrated polarimeter (GDx, Carl Zeiss Meditec). There is a considerable amount of scientific evidence about the role of this imaging technique for glaucoma diagnosis.^25^

Medeiros et al reported the ability of GDx variable corneal compensation (GDx VCC) to detect progressive RNFL loss using trend-based approach.^26^ The rate of decline was significantly higher in the progressing group (-0.70 mm/y) compared with the nonprogressing group (-0.14 mm/y, p = 0.001). They also found that black race and male sex were significantly associated with higher rates of RNFL loss during follow-up. In their subsequent publication using the updated version GDx enhanced corneal compensation (GDx ECC), they also reported the significant correlation between intraocular pressure and RNFL thickness reduction.^27^ In another report from the same institute, the rates of change in RNFL thickness measured with GDx ECC *vs* neuroretinal rim area measured with confocal scanning laser tomography (HRT, Heidelberg engineering) were compared.^28^ They reported that although average declination rate of RNFL thickness was significantly different between progressors and nonprogressors, rim area decline was not significantly different between groups. Most recently, Medeiros et al presented a new methodology for combining structural and functional tests to improve detection of glaucoma progression and estimation of rates of change.^29^ They reported that integrating structural (GDx ECC) and functional (SAP) information using Bayesian hierarchical models identified a significantly higher proportion of the glaucomatous and suspect eyes as having progressed when compared with the conventional ordinary least square method (22.7% *vs* 12.8%; p = 0.001) while having the same specificity of 100%.

### Confocal Scanning Laser Ophthalmoscopy

CSLO of the ONH has been available commercially more than 20 years ago using Heidelberg retina tomography (HRT). The HRT employs a diode laser (670 nm wavelength) to sequentially scan the retinal surface in the x and y directions at multiple focal planes. A three-dimensional topographic image is then constructed from a series of optical image sections at consecutive focal planes to provide retinal height measurements of the optic nerve head.^30,31^ The HRT has been used primarily to measure and characterize the ONH topography.

Kamal et al first reported the use of HRT to detect glaucoma progression in ocular hypertension (OHT) patients who had converted to early glaucoma on the basis of VF criteria.^32,33^ They demonstrated that many of the HRT parameters showed statistically significant change between baseline and follow-up visit using Wilcoxon signed rank test. Chauhan et al developed a technique called TCA (topographical change analysis) for detecting serial topographic changes in ONH.^34,35^ They divided original images into 64 × 64 superpixels and evaluated the significant change between baseline and follow-up examination in each superpixel using an analysis of variance technique. Using TCA, they compared the proportion of eyes that showed progression between HRT and SAP in patients with early glaucoma. A total of 40% of eyes showed progression with HRT only, 4% with VF only and 29% showed progression with both techniques. They also reported that longitudinally measured ONH change is predictive of subsequent VF progression using the same technique.^36^

Tan et al performed event-based analyses of ONH topography measurements.^37,38^ Progression was evaluated for each 30°sector of rim area based on the estimated measurement variability. They evaluated the ability of this method in OHT patients who converted to glaucoma and normal control subjects. A total of 85% of converters (sensitivity) and 5% of control subjects (false-positive responses) had positive test results. However, we should take into consideration that this very high sensitivity and specificity was acquired using somewhat biased population of progressors *vs* completely healthy controls which does not represents actual patient population.

Patterson et al reported another method called SIM (statistic image mapping) for detecting change in serial ONH topography images.^39^ SIM utilize the permutation testing strategy to detect significant change in each pixel. They show that SIM was superior to TCA in sensitivity (73% *vs* 53%) and specificity (90% *vs* 85%).

Strouthidis et al performed a trend-based analyses of rim area in patients with OHT.^40^ They used several different criteria of progression to compare the VF progression and HRT progression. They reported poor agreement between HRT and VF progression regardless of the progression criteria. Fayers et al described an event analysis for monitoring HRT progression and its specificity, detection rate and agreement with visual field progression.^41^ They used four distinct criteria for progression. In their cohort, it had a higher detection rate of progression than RA trend analysis and the VF progression criteria with fixed specificity. See et al found a more rapid decline of rim area measurements in glaucoma patients compared to control subjects using trend analyses of HRT measured rim area.^42^ Medeiros et al reported that combining structural (HRT) and functional (SAP) information with Bayesian method^29^ provided more accurate prediction of future glaucoma progression than conventional methods.^43^

## LIMITATIONS OF THE CURRENT EVIDENCE

### Performances of Imaging Instruments to Detect Progression

Performances of imaging instruments to detect progression reported in literature are summarized in Table 1. Although these studies show overall favorable performances of imaging instrument in detecting glaucoma progression, there is considerable inter- and intrainstrument variability. It is, at least in part, because of the variability in the method of analysis, sample population and the reference standard used in these studies. In many of these reports, sensitivity was determined in a group of patients showing distinct progression and specificity was determined in a separate group of apparently normal subjects. Although commonly used, these study population are quite dissimilar to the clinically relevant population. The performance of a test may be overestimated by clinical studies in these settings, known as spectrum bias.^44-46^ Another limitation in designing studies assessing glaucoma progression is the absence of a perfect reference standard.

**Table 1:** **Table 1:** Performances of imaging instruments to detect progression

*Authors*		*Instrument*		*Sensitivity*		*Specificity*		*AUC*		*Analysis*		*Sample*		*Reference standard*	
Lee et al^18^		Stratus		62.0%		80.0%		_―_		Trend		Glaucoma*		Red-free photography	
Lee et al^15^		Stratus		85.2%		59.7%		―		Event		Glaucoma		Red-free photography	
Alencar et al^28^		GDx		―		―		0.811		Trend		Glaucoma/ Glaucoma suspect		Stereophotography/SAP	
Alencar et al^28^		HRT		―		―		0.507		Trend		Glaucoma/ Glaucoma suspect		Stereophotography/SAP	
Fayers T et al^41^		HRT		94.1%		95.2%		―		Event		OHT		SAP (AGIS criteria)	
Patterson et al^39^		HRT		73.0%		90.0%		―		SIM		OHT converters		SAP (AGIS criteria)	
Tan et al^37^		HRT		90.0%		65.0%		―		Event		OHT converters		SAP (AGIS criteria)	
Tan et al^38^		HRT		90.0%		93.8%		―		Event		OHT converters		SAP (AGIS criteria)	

*Glaucoma with localized RNFL defect; AUC: Area under the curve; SAP: Standard automated perimetry; SIM: Statistic image mapping; AGIS: Advanced glaucoma intervention study

**Table 2:** **Table 2:** Agreement between structural and functional progression

*Authors*		*Instrument*		*Analysis*		*Progression with* *structure only*		*Progression with* *VF only*		*Progression with* *both*	
Wollstein et al^13^		Prototype OCT		Event		22.0%		9.0%		3.0%	
Leung et al^16^		Stratus		Trend		15.5%		16.4%		2.6%	
Leung et al^17^		Stratus		Trend		5.6%		28.7%		3.7%	
Leung et al^17^		HRT		Trend		8.3%		25.9%		6.5%	
Cauhan et al^35^		HRT		TCA		40.0%		19.0%		2.6%	
Strouthidis et al^40^		HRT		Trend		8.6%		15.1%		3.5%	

OCT: Optical coherence tomography; TCA: Topographical change analysis

There are few studies directly comparing the diagnostic ability to detect glaucoma progression obtained with the latest instruments in one study population.^17,19,28^ Moreover, the diagnostic accuracy of the measurements can vary by study population, so the results obtained in one study cannot be extrapolated to general population. At this moment, we can only conclude that the precision of the current imaging instruments is generally good, but there is no agreement regarding the most appropriate method for the evaluating structural progression.

### Agreement between Structural and Functional Progression

Table 2 summarizes correlations between structural and functional progression in glaucoma reported in literature. The agreement between structural and functional progression is generally poor, regardless of the instruments or analyses used to detect progression. The disagreement between structural and functional methods for detecting progression could be related to the different algorithms used to assess change, the variability of measurements over time or the different scales used to assess structure and function.^14,16,35^ In addition, it is believed that structural change precedes functional change during the course of glaucoma progression. More precisely, detectable structural change precedes detectable functional change. Previous studies have suggested that imaging devices may be better suited for detection of progression at relatively early stages of disease, whereas the technology may fail to detect change in cases with advanced damage.^26,27^ In contrast, the logarithmic scaling of clinical visual field measurements may favor detection of change in later stages of disease with SAP.^47,48^ These observations may indicate that structural examinations detect a different subset of patients with progression that cannot be detected with visual field examinations. Imaging devices may serve as useful adjuncts to provide complementary information rather than to replace functional examinations in the evaluation of glaucoma progression.

## CONCLUSION

1. Imaging technologies provide objective and quantitative analyses of glaucoma progression.
2. Both event and trend analyses are useful for change detection.
3. Rates of RNFL and rim area loss are significantly faster in progressing compared with non progressing subjects.
4. The precision of the current imaging instruments is generally good, but there is no uniform agreement regarding the most appropriate method for the evaluation of structural progression associated with glaucoma.
5. The agreement between structural and functional glaucoma progression is generally poor regardless of the strategies used.
6. Combining structural and functional analyses are necessary for accurate monitoring of glaucoma progression.
7. It is not recommended that isolated clinical decisions be based solely upon ocular imaging results. Clinical correlation should be performed and treatment recommendations should be individualized.

## References

[1] Weinreb RN, Khaw PT (2004). Primary open-angle glaucoma.. Lancet.

[2] Nouri-Mahdavi K, Nassiri N, Giangiacomo A, Caprioli J (2011). Detection of visual field progression in glaucoma with standard achromatic perimetry: A review and practical implications.. Graefes Arch Clin Exp Ophthalmol.

[3] Sommer A, Katz J, Quigley HA, Miller NR, Robin AL, Richter RC, Witt KA (1991). Clinically detectable nerve fiber atrophy precedes the onset of glaucomatous field loss.. Arch Ophthalmol.

[4] Quigley HA, Katz J, Derick RJ, Gilbert D, Sommer A (1992). An evaluation of optic disc and nerve fiber layer examinations in monitoring progression of early glaucoma damage.. Ophthalmology.

[5] Kass MA, Heuer DK, Higginbotham EJ, Johnson CA, Keltner JL, Miller JP, Parrish RK 2nd, Wilson MR, Gordon MO (2002). The Ocular Hypertension Treatment Study: A randomized trial determines that topical ocular hypotensive medication delays or prevents the onset of primary open-angle glaucoma.. Arch Ophthalmol.

[6] Miglior S, Zeyen T, Pfeiffer N (2005). Results of the European glaucoma prevention study.. Ophthalmology.

[7] Medeiros FA, Alencar LM, Zangwill LM, Bowd C, Sample PA, Weinreb RN (2009). Prediction of functional loss in glaucoma from progressive optic disc damage.. Arch Ophthalmol.

[8] Jampel HD, Friedman D, Quigley HA (2009). Agreement among glaucoma specialists in assessing progressive disc changes from photographs in open-angle glaucoma patients.. Am J Ophthalmol.

[9] Greenfield DS (2002). Optic nerve and retinal nerve fiber layer analyzers in glaucoma.. Curr Opin Ophthalmol.

[10] Townsend KA, Wollstein G, Schuman JS (2009). Imaging of the retinal nerve fibre layer for glaucoma.. Br J Ophthalmol.

[11] Sung KR, Kim JS, Wollstein G, Folio L, Kook MS, Schuman JS (2011). Imaging of the retinal nerve fibre layer with spectral domain optical coherence tomography for glaucoma diagnosis.. Br J Ophthalmol.

[12] Huang D, Swanson E, Lin C (1991). Optical coherence tomography.. Science.

[13] Wollstein G, Schuman JS, Price LL (2005). Optical coherence tomography longitudinal evaluation of retinal nerve fiber layer thickness in glaucoma.. Arch Ophthalmol.

[14] Medeiros FA, Zangwill LM, Alencar LM (2009). Detection of glaucoma progression with stratus OCT retinal nerve fiber layer, optic nerve head, and macular thickness measurements.. Invest Ophthalmol Vis Sci.

[15] Lee EJ, Kim TW, Park KH, Seong M, Kim H, Kim DM (2009). Ability of Stratus OCT to detect progressive retinal nerve fiber layer atrophy in glaucoma.. Invest Ophthalmol Vis Sci.

[16] Leung CKS, Cheung CYL, Weinreb RN (2010). Evaluation of retinal nerve fiber layer progression in glaucoma: A study on optical coherence tomography guided progression analysis.. Invest Ophthalmol Vis Sci.

[17] Leung CKS, Liu S, Weinreb RN (2011). Evaluation of retinal nerve fiber layer progression in glaucoma: A prospective analysis with neuroretinal rim and visual field progression.. Ophthalmology.

[18] Lee EJ, Kim TW, Weinreb RN, Park KH, Kim SH, Kim DM (2011). Trend-based analysis of retinal nerve fiber layer thickness measured by optical coherence tomography in eyes with localized nerve fiber layer defects.. Invest Ophthalmol Vis Sci.

[19] Leung CKS, Chiu V, Weinreb RN (2011). Evaluation of retinal nerve fiber layer progression in glaucoma: A comparison between spectral-domain and time-domain optical coherence tomography.. Ophthalmology.

[20] Tan O, Chopra V, Lu AT-H, Schuman JS (2009). Detection of macular ganglion cell loss in glaucoma by Fourier-domain optical coherence tomography.. Ophthalmology.

[21] Kiernan DF, Mieler WF, Hariprasad SM (2010). Spectral-domain optical coherence tomography: A comparison of modern high-resolution retinal imaging systems.. Am J Ophthalmol.

[22] Mwanza JC, Oakley JD, Budenz DL, Chang RT, Knight OJ, Feuer WJ (2011). Macular ganglion cell-inner plexiform layer: Automated detection and thickness reproducibility with spectral domain-optical coherence tomography in glaucoma.. Invest Ophthalmol Vis Sci.

[23] Weinreb RN (1999). Evaluating the retinal nerve fiber layer in glaucoma with scanning laser polarimetry.. Arch Ophthalmol.

[24] Weinreb RN, Dreher AW, Coleman A, Quigley H, Shaw B, Reiter K (1990). Histopathologic validation of Fourier-ellipsometry measurements of retinal nerve fiber layer thickness.. Arch Ophthalmol.

[25] Pozzo SD, Marchesan R, Ravalico G (2009). Scanning laser polarimetry―a review.. Clin Experiment Ophthalmol.

[26] Medeiros FA, Alencar LM, Zangwill LM (2009). Detection of progressive retinal nerve fiber layer loss in glaucoma using scanning laser polarimetry with variable corneal compensation.. Invest Ophthalmol Vis Sci.

[27] Medeiros FA, Alencar LM, Zangwill LM, Sample PA, Weinreb RN (2008). The relationship between intraocular pressure and progressive retinal nerve fiber layer loss in glaucoma.. Ophthalmology.

[28] Alencar LM, Zangwill LM, Weinreb RN (2010). A comparison of rates of change in neuroretinal rim area and retinal nerve fiber layer thickness in progressive glaucoma.. Invest Ophthalmol Vis Sci.

[29] Medeiros FA, Leite MT, Zangwill LM, Weinreb RN (2011). Combining structural and functional measurements to improve detection of glaucoma progression using Bayesian hierarchical models.. Invest Ophthalmol Vis Sci.

[30] Zangwill LM, Bowd C (2006). Retinal nerve fiber layer analysis in the diagnosis of glaucoma.. Curr Opin Ophthalmol.

[31] Mansouri K, Leite MT, Medeiros FA, Leung CKS, Weinreb RN (2011). Assessment of rates of structural change in glaucoma using imaging technologies.. Eye.

[32] Kamal DS, Viswanathan AC, Hitchings RA, Poinoosawmy D (1999). Detection of optic disc change with the Heidelberg retina tomograph before confirmed visual field change in ocular hypertensives converting to early glaucoma.. Br J Ophthalmol.

[33] Kamal DS, Hitchings RA, Fitzke FW (2000). Use of sequential Heidelberg retina tomograph images to identify changes at the optic disc in ocular hypertensive patients at risk of developing glaucoma.. Br J Ophthalmol.

[34] Chauhan BC, Blanchard JW, Hamilton DC, Leblanc RP (2000). Technique for detecting serial topographic changes in the optic disc and peripapillary retina using scanning laser tomography.. Invest Ophthalmol Vis Sci.

[35] Chauhan BC, McCormick B, Nicolela MT, LeBlanc RP (2002). Optic disc and visual field changes in a prospective longitudinal study of patients with glaucoma: Comparison of scanning laser tomography with conventional perimetry and optic disc photography.. Arch Ophthalmol.

[36] Chauhan BC, Nicolela MT, Artes PH (2009). Incidence and rates of visual field progression after longitudinally measured optic disc change in glaucoma.. Ophthalmology.

[37] Tan JCH, Hitchings RA (2003). Approach for Identifying Glaucomatous Optic Nerve Progression by Scanning Laser Tomography.. Invest Ophthalmol Vis Sci.

[38] Tan JCH, Hitchings RA (2004). Optimizing and validating an approach for identifying glaucomatous change in optic nerve topography.. Invest Ophthalmol Vis Sci.

[39] Patterson AJ, Garway-Heath DF, Strouthidis NG, Crabb DP (2005). A new statistical approach for quantifying change in series of retinal and optic nerve head topography images.. Invest Ophthalmol Vis Sci.

[40] Strouthidis NG, Scott A, Peter NM, Garway-Heath DF (2006). Optic disc and visual field progression in ocular hypertensive subjects: Detection rates, specificity, and agreement.. Invest Ophthalmol Vis Sci.

[41] Fayers T, Strouthidis NG, Garway-Heath DF (2007). Monitoring glauco-matous progression using a novel Heidelberg Retina Tomograph event analysis.. Ophthalmology.

[42] See JLS, Nicolela MT, Chauhan BC (2009). Rates of neuroretinal rim and peripapillary atrophy area change: A comparative study of glaucoma patients and normal controls.. Ophthalmology.

[43] Medeiros FA, Zangwill LM, Girkin CA (2012; In press). Combining structural and functional measurements to improve estimates of rates of glaucomatous progression.. Am J Ophthalmol.

[44] Ransohoff DF, Feinstein AR (1978). Problems of spectrum and bias in evaluating the efficacy of diagnostic tests.. N Engl J Med.

[45] Lijmer JG, Mol BW, Heisterkamp S (1999). Empirical evidence of design-related bias.. JAMA.

[46] Medeiros FA, Ng D, Zangwill LM, Sample PA, Bowd C, Weinreb RN (2007). The effects of study design and spectrum bias on the evaluation of diagnostic accuracy of confocal scanning laser ophthalmoscopy in glaucoma.. Invest Ophthalmol Vis Sci.

[47] Garway-Heath DF, Caprioli J, Fitzke FW, Hitchings RA (2000). Scaling the Hill of Vision: The physiological relationship between light sensitivity and ganglion cell numbers.. Invest Ophthalmol Vis Sci.

[48] Hood DC, Kardon RH (2007). A framework for comparing structural and functional measures of glaucomatous damage.. Prog Retin Eye Res.

